# Mischievous responding in Internet Gaming Disorder research

**DOI:** 10.7717/peerj.2401

**Published:** 2016-09-13

**Authors:** Andrew K. Przybylski

**Affiliations:** Oxford Internet Institute, University of Oxford, Oxford, United Kingdom

**Keywords:** Internet-based games, Mischievous responding, Internet Gaming Disorder, Computer networks & communications, Video games, Internet, Serious games, Addiction, Web, computer games

## Abstract

The most recent update to the American Psychiatric Association’s (APA) Diagnostic and Statistical Manual of Mental Disorders (DSM-5) included Internet Gaming Disorder as a new potential psychiatric condition that merited further scientific study. The present research was conducted in response to the APA Substance-Related Disorders Working Group’s research call to estimate the extent to which mischievous responding—a known problematic pattern of participant self-report responding in questionnaires—is relevant to Internet Gaming Disorder research. In line with a registered sampling and analysis plan, findings from two studies (*n*_tot_ = 11,908) provide clear evidence that mischievous responding is positively associated with the number of Internet Gaming Disorder indicators participants report. Results are discussed in the context of ongoing problem gaming research and the discussion provides recommendations for improving the quality of scientific practice in this area.

## Introduction

Internet-based games are now a dominant form of entertainment for many as advances in digital technology enable play on a variety of devices ranging from powerful gaming computers to ubiquitous smartphones (Lenhart, 2015; Duggan, 2015). Internet-based games allow millions to connect and play in both computer mediated environments like World of Warcraft and engage with increasingly popular augmented reality games such as Pokémon GO (Chess, 2014). Such games integrate Internet data with real time visual and location information to layer gameplay over everyday contexts. The widespread popularity of Internet-based games have given rise to concerns they may have negative effects and may be behaviorally dysregulating (American Psychiatric Association, 2013). Responding to this call, the American Psychiatric Association’s Substance-Related Disorders working group highlighted the need for basic scientific research to investigate whether Internet Gaming Disorder (IGD) is a psychiatric condition akin to Gambling Disorder (Hasin et al., 2013).

There is a growing literature examining problematic gaming (Petry et al., 2014; Kardefelt-Winther, 2014; Griffiths et al., 2016). Although most of this work does not distinguish between games that do and do not integrate Internet technology, the theoretical and empirical work on IGD is advancing at a rapid pace (King et al., 2013). Meta-analytic and conceptual research suggests that problem gaming may indeed merit serious clinical attention and caution methodological hurdles currently hinder a full empirical understanding of IGD (Ferguson, Coulson & Barnett, 2011; Kardefelt-Winther, 2014). For example, the use of convenience samples and a reliance on paper and computer-based self-report methodologies challenge the robustness of the literature. As a result estimates of the problem gaming vary widely, from as low as 0.2% (Festl, Scharkow & Quandt, 2013) to as high as 45% (Wan & Chiou, 2006). Work to address these challenges has been relatively slow in coming the literature has not started to grapple with issues known to plague health research more broadly.

Because nearly all problem gaming data are collected via paper or digitally distributed questionnaires substantial attention has been paid to the development of self-report instruments. By one estimate, at least 85 scales have been used to measure problem gaming yet there are relatively few careful studies that evaluate the underlying psychometric assumptions used in more than a single study (King et al., 2013). This lack of follow-up is noteworthy because it leaves many important questions, such as the reliability and truthfulness of responders, unexamined when drawing inferences about the prevalence and effects of IGD. Given the high clinical stakes, namely the real possibility of being treated as a recognized psychiatric disorder, it is important to scrutinize the quality of data provided through self-report and ensure the methodologies used are themselves valid.

Findings from health research indicates that many as 2% of those responding to mental and physical well-being screening questionnaires respond in a problematic way (Furlong, Fullchange & Dowdy, in press), and nearly 10% of respondents to health and substance use questionnaires claim to have knowledge of a fictional recreational drug (Fuller & Hawkins, 2012). Research indicates such “jokesters,” labeled by researchers as mischievous responders, do in fact provide extreme and untruthful responses that can dramatically effect point estimates for uncommon, yet important, phenomena such as variability in gender identity (Robinson-Cimpian, 2014). For example 40% of supposed transgender identifying participants were flagged by analyses sensitive to mischievously responding compared to only 1.5% of cisgender participants. Participant misrepresentation through self-report can be dramatic, one study examining this responding pattern found that nearly all of participants (99%) who reported having an artificial limb did not in fact have one when they were subsequently interviewed in person (Fan et al., 2006). When one considers the relatively low likely prevalence of problem gaming, the extant literature’s reliance on self-report questionnaires, and parallel evidence in similar research domains there is good reason to rigorously investigate the relevance of mischievous responding to IGD research.

### The present research

In line with this, the purpose of the present study was to evaluate the idea that mischievous participant responding could be an important factor to consider in problem gaming research. It was predicted that mischievous responding would serve to inflate the acute period prevalence estimates of IGD indicators. Because of the high-stakes for clinical practice, the present research adopted a rigorous open science approach wherein the sampling and analysis plans were registered in advance of data collection.

## Method

### Participants and measures

Data from two samples part of a larger project on Internet Gaming Disorder (Przybylski, Weinstein & Murayama, in press) were analyzed. Study 1 was comprised of a cohort of adults aged 18 years and older from the United Kingdom (941 females, 958 males), and Study 2 was composed of four young adult cohorts aged 18–24 years from the United States, United Kingdom, Canada, and Germany (4,995 females, 5,014 males). All participants were recruited through Google surveys between April and June 2015 using joint distributions of age, gender, and geographic location information inferred from web tracking data. This data collection method was used because it presented a low participant burden, overall survey completion rate was relatively high (80.3%), and past research has demonstrated the utility of using Google surveys for large scale data collection (e.g., McDonald, Mohebbi & Slatkin, 2012; Sell, Goldberg & Conron, 2015). The research presented minimal risk, and was granted clearance by the ethics committee of the Oxford Internet Institute at the University of Oxford (CUREC/C1A15-006). The analysis plans for both studies were registered in advance of data collection (Przybylski, 2016a; Przybylski, 2016b) and both data and code are available for download using the Open Science Framework (Przybylski, 2016c).

#### Internet Gaming Disorder

In line DSM-5 guidance participants completed an indicator checklist to measure Internet Gaming Disorder. Individual indicators could be either not be endorsed (coded 0) or endorsed (coded 1) and individual indicator counts were created by summing the number of indicators. Because the number of indicators reported decreased monotonically in both samples the average number indicators endorsed in both Study 1 (*M* = 0.68, *α* = .76) and Study 2 (*M* = 0.73, *α* = .72) were relatively low. For example, in Study 2, 66.5% of participants reported no indicators, 14.9% reported a single indicator, 10.7% reported two, 3.3% reported three, 2.0% reported four, 1.2% reported five, 0.7% reported six, 0.3% reported seven, and 0.4% reported eight indicators.

#### Mischievous responding

To assess problematic responding (Robinson-Cimpian, 2014) a sham item; “In the past year I have played the game Semeron Online” was used to check if participants were excessively or carelessly selecting indicators during self-report. Because Semeron Online is the title of a fictitious game participants could not truthfully report that they had recently played the game. Those who reported recently playing it were coded as mischievous (1) whereas those who did not were coded 0. In Study 1 the prevalence of mischievous responding was 1.47% (95% CI [0.95%–2.00%]), and in in Study 2 it was 2.27% (95% CI [1.99%–2.57%]).

## Results

### Exploratory analyses

Zero-order correlation matrices for both studies are presented in Table 1. Results from this exploratory analysis indicated that gender was modestly related to both mischievous responding and the number of IGD indicators reported (all *ps* < .001). In both cases, males reported slightly higher levels.

**Table 1:** Observed Zero-order Correlations.

	1.	2.	3.
1. Gender	–	-0.01	0.04
2. Mischievous responding	0.03^**^	–	0.10^***^
3. IGD indicators reported	0.04^***^	0.10^***^	–

**Notes.**

Study 1 coefficients above the diagonal. Study 2 coefficients below the diagonal.

** *p* < .001.

*** *p* < .01.

### Confirmatory analyses

**Figure 1:**
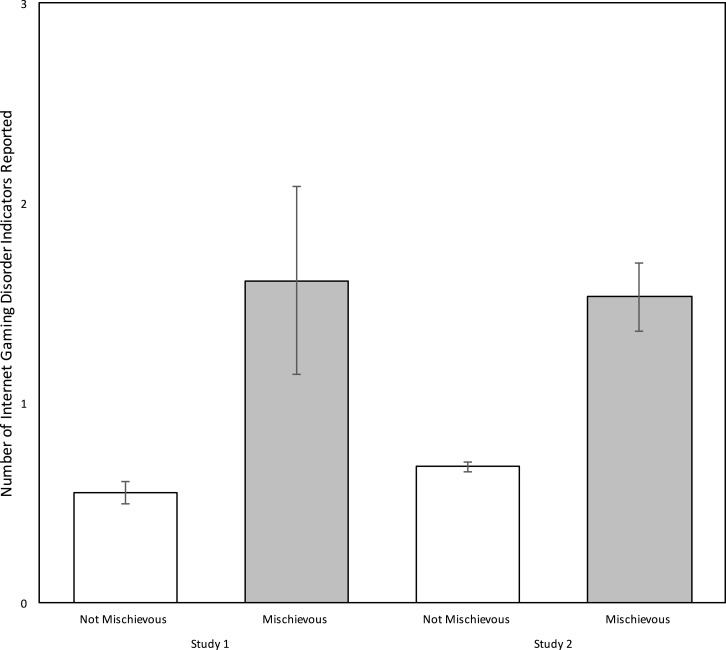
Number of IGD indicators reported by not mischievous and mischievous responders. *Note*. Error bars denote the 95% confidence interval for the observed means. Both comparisons between Not Mischievous to Mischievous responders were statistically significant at the *p* < 0.001 level.

It was hypothesized, a priori, that mischievous responding would be positively associated with the number of Internet Gaming Disorder indicators participants would report. Results from two planned analyses in Study 1, *F*(1,1897) 19.82, *p* < .0001, and in Study, *F*(1,10007) 100.17, *p* < .0001, supported the prediction. An examination of the means for non-mischievous and mischievous responders, presented in Fig. 1, indicated the later tended to report a greater number of indicators in both Study 1, *M* = 1.61 (95% CI [1.15–2.07]) vs. *M* = 0.55 (95% CI [0.50–0.61]) and Study 2, *M* = 1.53 (95% CI [1.37–1.70]), vs. *M* = 0.68 (95% CI [0.65–0.70]). In both studies the observed effect sizes, Cohen’s *d*, reflecting the association between mischievous responding and Internet Gaming Disorder indicators were modest yet consistent, *d* = 0.204 in Study 1, and *d* = 0.200 in Study 3.

## Discussion

The scientific study of Internet Gaming Disorder is at an early stage and there are active debates regarding the existence and nature of the proposed phenomenon (Petry et al., 2014; Kardefelt-Winther, 2014; Griffiths et al., 2016). Given that problem gaming it is seriously being considered for inclusion as psychiatric disorder in future revisions of the DSM transparent and robust evidence is needed in order for scientists to comprehensively vet the its measurement, etiology, prevalence, stability, and consequences. The present research aimed and succeeded in providing a reliable insight into the problem gaming research: IGD, as assessed through self-report, can be inflated by mischievous responding. By following a registered sampling and analysis plan, the findings show IGD measurement is vulnerable to a phenomenon known to effect self-report based research in other health research domains. Results from two large-scale studies indicated between 1.47% and 2.27% of participants responded mischievously and confirmed between that this pattern of excessive or careless self-reporting is associated with responding to the clinical checklist indicators proposed in the DSM-5. Because the present results indicate that IGD measurement at levels observed in other questionnaire-based health research (e.g., Furlong, Fullchange & Dowdy, in press), this finding should be integrated directly into ongoing IGD research. Future studies examining IGD should include robust diagnostic checks for mischievous responding and correct for its effects when they are found (Robinson-Cimpian, 2014). Without these corrections it is possible that future research on this potential disorder could overestimate its prevalence and exaggerate its clinical impact.

### Limitations

The present research presents three important limitations that suggest promising directions for future research. First, the present research depended solely on data provided through individual self-reports. Convergent data from multiple respondents, for example friends, caregivers, or romantic partners, are needed in IGD research. Such data would add greatly to our understanding of the development and prevalence of this phenomenon (Cronbach & Meehl, 1955). Second, a range of alternative methods have been used to operationalize mischievous responding and it is possible that attention checks and quantifying extremes in responding may be more effective than the approach used in the present study. Alternative approaches for detecting and mitigating the influence of this problematic pattern should be studied (Robinson-Cimpian, 2014). Finally, the present research utilized a checklist-based approach for assessing the relations between mischievous responding and IGD measurement. Although the checklist approach is implicit to the DSM-5, guidance many studying IGD have elected to use alternate methods (Hasin et al., 2013; Petry et al., 2014; Griffiths et al., 2016). It will be important for future research to evaluate the extent to which mischievous responding impacts these graded measurement approaches.

### Closing remarks

Internet-based gaming is currently one of the most popular recreational activities in the developed and developing world (Lenhart, 2015). Because this activity is ubiquitous, researchers studying its potential downsides must hold themselves to the highest scientific standards available in the social and clinical sciences. Testing the influence of mischievous responding, as was done in the present studies, provides a single necessary empirical step in this pursuit. This research registered its hypothesis in advance of data collection and its data and code open by default (Przybylski, 2016a; Przybylski, 2016b; Przybylski, 2016c). A great deal of work remains to be done. Given the high reputational and clinical stakes for this research area, studies on IGD must use both open data and materials (Elson, Przybylski & Krämer, 2015; Morey et al., 2016). Only open and rigorous scientific methodologies will be able to meaningfully elucidate this potentially important phenomenon. This research area is at a formative stage and a critical understanding of the nature, prevalence, and etiology of Internet Gaming Disorder must be based in rigorous and open methodologies before conclusions are drawn and policy is made.

##  Supplemental Information

10.7717/peerj.2401/supp-1 Data S1 Study 1 data

10.7717/peerj.2401/supp-2 Data S2 Study 2 data

10.7717/peerj.2401/supp-3 Supplemental Information 1 Code and analyses

## Additional Information and Declarations

### Data Availability

The following information was supplied regarding data availability: The Open Science Framework: https://osf.io/wvezd/?view_only=f51dd8f476ac409a9e2738a9fae4dcf2.
