# Supplementary material for: Mischievous responding in Internet Gaming Disorder research
Source: PeerJ. 2016 Sep 13;4:e2401. doi: 10.7717/peerj.2401 (PMC5028771; doi:10.7717/peerj.2401)
Supplement: Data S1 [file peerj-04-2401-s001.jasp › index.html]

JASP 


# Results

## Descriptives

| Descriptive Statistics | | | |
| --- | --- | --- | --- |
|  | | sex | |
| Valid |  | 1899 |  |
| Missing |  | 0 |  |
|  | | | |

### Frequencies

| Frequencies for sex | | | | | | | | | |
| --- | --- | --- | --- | --- | --- | --- | --- | --- | --- |
|  | | Frequency | | Percent | | Valid Percent | | Cumulative Percent | |
| 0 |  | 958 |  | 50.4 |  | 50.4 |  | 50.4 |  |
| 1 |  | 941 |  | 49.6 |  | 49.6 |  | 100.0 |  |
| Total |  | 1899 |  | 100.0 |  | 100.0 |  |  |  |
|  | | | | | | | | | |

## Reliability Analysis

| Scale Reliability Statistics | | | |
| --- | --- | --- | --- |
|  | | Cronbach's α | |
| scale |  | 0.764 |  |
|  | | | |
|  |  |  |  |
| --- | --- | --- | --- |
| *Note.*  Scale consists of items preoccupation, withdrawl, tolerance, nocontrol, continuing, misleading, escaping, givingup, risking | | | |

## Descriptives

| Descriptive Statistics | | | |
| --- | --- | --- | --- |
|  | | mischievous | |
| Valid |  | 1899 |  |
| Missing |  | 0 |  |
| Mean |  | 0.01474 |  |
|  | | | |

### Frequencies

| Frequencies for mischievous | | | | | | | | | |
| --- | --- | --- | --- | --- | --- | --- | --- | --- | --- |
|  | | Frequency | | Percent | | Valid Percent | | Cumulative Percent | |
| 0 |  | 1871 |  | 98.5 |  | 98.5 |  | 98.5 |  |
| 1 |  | 28 |  | 1.5 |  | 1.5 |  | 100.0 |  |
| Total |  | 1899 |  | 100.0 |  | 100.0 |  |  |  |
|  | | | | | | | | | |

## Correlation Matrix

| Pearson Correlations | | | | | | | | | |
| --- | --- | --- | --- | --- | --- | --- | --- | --- | --- |
|  | |  | | sex | | mischievous | | indicator\_count | |
| sex |  | Pearson's r |  | — |  | -0.008 |  | 0.039 |  |
|  |  | p-value |  | — |  | 0.739 |  | 0.092 |  |
| mischievous |  | Pearson's r |  |  |  | — |  | 0.102 |  |
|  |  | p-value |  |  |  | — |  | < .001 |  |
| indicator\_count |  | Pearson's r |  |  |  |  |  | — |  |
|  |  | p-value |  |  |  |  |  | — |  |
|  | | | | | | | | | |

## ANOVA

| ANOVA - indicator\_count | | | | | | | | | | | | | |
| --- | --- | --- | --- | --- | --- | --- | --- | --- | --- | --- | --- | --- | --- |
| Cases | | Sum of Squares | | df | | Mean Square | | F | | p | | η² | |
| mischievous |  | 30.58 |  | 1 |  | 30.583 |  | 19.82 |  | < .001 |  | 0.010 |  |
| Residual |  | 2926.92 |  | 1897 |  | 1.543 |  |  |  |  |  |  |  |
|  | | | | | | | | | | | | | |
|  |  |  |  |  |  |  |  |  |  |  |  |  |  |
| --- | --- | --- | --- | --- | --- | --- | --- | --- | --- | --- | --- | --- | --- |
| *Note.*  Type III Sum of Squares | | | | | | | | | | | | | |

### Marginal Means

| Marginal Means - mischievous | | | | | | | | | |
| --- | --- | --- | --- | --- | --- | --- | --- | --- | --- |
| mischievous | | Marginal Mean | | SE | | Lower CI | | Upper CI | |
| 0 |  | 0.554 |  | 0.029 |  | 0.498 |  | 0.611 |  |
| 1 |  | 1.607 |  | 0.235 |  | 1.147 |  | 2.068 |  |
|  | | | | | | | | | |
