# Supplementary material for: Mischievous responding in Internet Gaming Disorder research
Source: PeerJ. 2016 Sep 13;4:e2401. doi: 10.7717/peerj.2401 (PMC5028771; doi:10.7717/peerj.2401)
Supplement: Data S2 [file peerj-04-2401-s002.jasp › index.html]

JASP 


# Results

## Descriptives

| Descriptive Statistics | | | |
| --- | --- | --- | --- |
|  | | Gender | |
| Valid |  | 10009 |  |
| Missing |  | 0 |  |
|  | | | |

### Frequencies

| Frequencies for Gender | | | | | | | | | |
| --- | --- | --- | --- | --- | --- | --- | --- | --- | --- |
|  | | Frequency | | Percent | | Valid Percent | | Cumulative Percent | |
| Female |  | 4995 |  | 49.9 |  | 49.9 |  | 49.9 |  |
| Male |  | 5014 |  | 50.1 |  | 50.1 |  | 100.0 |  |
| Total |  | 10009 |  | 100.0 |  | 100.0 |  |  |  |
|  | | | | | | | | | |

## Reliability Analysis

| Scale Reliability Statistics | | | |
| --- | --- | --- | --- |
|  | | Cronbach's α | |
| scale |  | 0.718 |  |
|  | | | |
|  |  |  |  |
| --- | --- | --- | --- |
| *Note.*  Scale consists of items preoccupation, withdrawl, tolerance, nocontrol, continuing, misleading, escaping, givingup, risking, distress | | | |

## Descriptives

| Descriptive Statistics | | | |
| --- | --- | --- | --- |
|  | | mischievous | |
| Valid |  | 10009 |  |
| Missing |  | 0 |  |
|  | | | |

### Frequencies

| Frequencies for mischievous | | | | | | | | | |
| --- | --- | --- | --- | --- | --- | --- | --- | --- | --- |
|  | | Frequency | | Percent | | Valid Percent | | Cumulative Percent | |
| 0 |  | 9782 |  | 97.7 |  | 97.7 |  | 97.7 |  |
| 1 |  | 227 |  | 2.3 |  | 2.3 |  | 100.0 |  |
| Total |  | 10009 |  | 100.0 |  | 100.0 |  |  |  |
|  | | | | | | | | | |

## Correlation Matrix

| Pearson Correlations | | | | | | | | | |
| --- | --- | --- | --- | --- | --- | --- | --- | --- | --- |
|  | |  | | sex | | mischievous | | indicator\_count | |
| sex |  | Pearson's r |  | — |  | 0.034 |  | 0.040 |  |
|  |  | p-value |  | — |  | < .001 |  | < .001 |  |
| mischievous |  | Pearson's r |  |  |  | — |  | 0.100 |  |
|  |  | p-value |  |  |  | — |  | < .001 |  |
| indicator\_count |  | Pearson's r |  |  |  |  |  | — |  |
|  |  | p-value |  |  |  |  |  | — |  |
|  | | | | | | | | | |

## ANOVA

| ANOVA - indicator\_count | | | | | | | | | | | | | |
| --- | --- | --- | --- | --- | --- | --- | --- | --- | --- | --- | --- | --- | --- |
| Cases | | Sum of Squares | | df | | Mean Square | | F | | p | | η² | |
| mischievous |  | 163.3 |  | 1 |  | 163.290 |  | 100.2 |  | < .001 |  | 0.010 |  |
| Residual |  | 16312.0 |  | 10007 |  | 1.630 |  |  |  |  |  |  |  |
|  | | | | | | | | | | | | | |
|  |  |  |  |  |  |  |  |  |  |  |  |  |  |
| --- | --- | --- | --- | --- | --- | --- | --- | --- | --- | --- | --- | --- | --- |
| *Note.*  Type III Sum of Squares | | | | | | | | | | | | | |

### Marginal Means

| Marginal Means - mischievous | | | | | | | | | |
| --- | --- | --- | --- | --- | --- | --- | --- | --- | --- |
| mischievous | | Marginal Mean | | SE | | Lower CI | | Upper CI | |
| 0 |  | 0.675 |  | 0.013 |  | 0.650 |  | 0.700 |  |
| 1 |  | 1.533 |  | 0.085 |  | 1.367 |  | 1.699 |  |
|  | | | | | | | | | |
